# Antimicrobial Resistant Factors in *Klebsiella pneumoniae* Strains Isolated From Urinary Tract Infections, Wound Infections, Hospital Wastewater, and Cervical Cancers From Ghana, Togo, and Benin

**DOI:** 10.1155/ijog/5079377

**Published:** 2026-01-22

**Authors:** Biigba Yakubu

**Affiliations:** ^1^ Department of Biochemistry and Biotechnology, KNUST, Kumasi, Ghana, knust.edu.gh

**Keywords:** antimicrobial, factors, genetic, island, *Klebsiella pneumoniae*, pathogenicity, reading frames, recombination, resistance

## Abstract

*Klebsiella pneumoniae* is a Gram‐negative, facultatively anaerobic member of the Enterobacteriaceae that functions both as a gut commensal and a major opportunistic pathogen implicated in severe hospital and community‐acquired infections. The rapid global expansion of antimicrobial‐resistant *K. pneumoniae* lineages, particularly ESBL‐ and carbapenemase‐producing strains, poses an escalating public health threat by eroding available treatment options. This study investigated the genomic architecture and resistance mechanisms of *K. pneumoniae* isolates recovered from urinary tract infections, wound infections, and cervical cancer cases across Ghana, Togo, and Benin. Eight isolates were subjected to antimicrobial susceptibility profiling and whole genome sequencing using the Illumina MiSeq platform after DNA extraction via the Zymo protocol. Comprehensive genomic analyses including MLST, resistance gene detection (Abricate), phylogenetic reconstruction (iTOL), genomic island prediction (IslandViewer), genome structural analysis (Proksee), and statistical interrogation in R (v4.4.0) were performed to characterize genetic diversity and identify determinants of antimicrobial resistance. The isolates exhibited heterogeneous but overlapping resistance profiles, extensive carriage of AMR genes, and the presence of multiple genomic islands enriched for integrases, transposases, and antibiotic resistance cassettes. MLST and SNP‐based comparisons revealed both clonal clusters and genetically divergent lineages, while recombination analysis indicated mutation‐driven evolution with lineage‐specific recombination hotspots. Conserved gene orientation patterns and regions of atypical GC content further suggested historical acquisition of mobile genetic elements, including plasmid integrations and resistance islands. Collectively, these findings demonstrate the high genomic plasticity, multidrug‐resistant phenotypes, and dynamic evolutionary processes shaping *K. pneumoniae* populations circulating in West Africa. The study underscores the urgent need for continuous regional genomic surveillance to guide treatment policies and limit the further dissemination of high‐risk AMR clones.

## 1. Introduction

*Klebsiella pneumoniae* is a Gram‐negative, facultatively anaerobic, rod‐shaped bacterium that belongs to the Enterobacteriaceae family [1]. This bacterium is ubiquitously present in various environments, including soil, water, sewage, and the intestinal tracts of humans and animals. While *K. pneumoniae* is a natural component of the gut flora, it can act as an opportunistic pathogen, particularly in individuals with compromised immune systems [2].

*K. pneumoniae* is notably associated with nosocomial infections, which are infections acquired in hospital settings. These infections encompass a wide range of conditions, such as urinary tract infections, respiratory tract infections, bacteremia, septicemia, wound infections, and endocarditis [3]. The bacterium’s ability to persist on medical equipment and surfaces in healthcare environments makes it a significant contributor to hospital outbreaks [4].

One of the critical challenges posed by *K. pneumoniae* is its intrinsic resistance to multiple antibiotics, including first‐generation cephalosporins [5]. Moreover, this bacterium has a remarkable capacity to acquire additional resistance mechanisms, such as extended‐spectrum *β*‐lactamases and carbapenems [6]. These resistance traits complicate the treatment of infections caused by *K. pneumoniae*, making it a formidable pathogen in clinical settings.

The adaptability and resistance patterns of *K. pneumoniae* have led to its classification as a serious public health threat [7]. Effective infection control measures and prudent antibiotic stewardship are essential to prevent the spread of this bacterium in healthcare environments. Recent reports have highlighted the emergence of resistance in *K. pneumoniae* against several antibiotics, including those recently recommended by the World Health Organization (WHO).

Additionally and with respect to virulent factors, *K. pneumoniae* produces lipopolysaccharides and outer membrane proteins, which help in the evasion of the host immune system [8]. The presence of capsular polysaccharides equally enhances resistance to phagocytosis. Besides, genes encoding beta‐lactamases such as *blaAmpC* and *blaKPC* contribute to antibiotic resistance, making infections difficult to treat. These virulence determinants collectively enable *K. pneumoniae* to establish infections, persist in fertile environments, and resist antimicrobial therapies [9].

Given the growing concern over antibiotic resistance in most bacterial species [10], this study is aimed at exploring the antimicrobial resistance factors in *K. pneumoniae* species isolated from urinary and wound infections in Ghana, Togo, and Benin by examining the genetic features that enable the bacterial strains to resist antibiotics. The study also seeks to identify potential variations in antibiotic‐resistant patterns alongside the antibiotic‐resistant hotspots such as pathogenicity islands (PAIs) and recombination events. This research is crucial for developing targeted strategies to combat the spread of antibiotic‐resistant *K. pneumoniae* strains and improve patient outcomes in healthcare environments.

## 2. Methods

Eight *K. pneumoniae* isolates in this study were sampled from diverse sources including people with urinary tract infections, wound infections, cervical cancers, and hospital wastewater. They were subjected to an antibiotic sensitivity test using the Kirby–Bauer disk diffusion susceptibility test protocol [11] and the following antibiotics: ceftriaxone, cefotaxime, gentamicin, amikacin, chloramphenicol, meropenem, imipenem, piperacillin–tazobactam, ciprofloxacin, ofloxacin, levofloxacin, cotrimoxazole (trimethoprim + sulfamethoxazole), vancomycin, polymyxin B, tetracycline, and ampicillin. DNA from the confirmed antibiotic‐resistant samples was extracted using Zymo DNA extraction kits [12]. The extracted DNA was fragmented into smaller pieces, typically around 200–600 base pairs in length. This was done through enzymatic fragmentation using restriction enzymes. The next step was end repair and A‐tailing, where the fragmented DNA ends were repaired to create blunt ends using a combination of enzymes that filled in or removed overhangs. An A‐base was added to the 3 ^′^ end of each DNA fragment, creating an overhang that facilitated adapter ligation in the next step.

NanoDrop 2000 (Thermo Fisher, United States) was used to determine the concentration of the genomic DNA, and the integrity was checked by agarose gel electrophoresis. The genomic DNA was then screened for whole genome sequencing using two criteria: First, its OD260/280 was assessed in the range between 1.8 and 2.0, and second, any visual RNA contamination present in the DNA was checked. For library preparation, approximately 100 ng DNA was used, followed by paired‐end sequencing using the NextSeq DNA Library preparation kit (Illumina, Inc., United States). Sequencing was done using the high‐throughput Illumina NextSeq sequencing platform (Illumina, Inc., United States).

Raw Illumina MiSeq reads were subjected to initial quality assessment using FastQC, and summary quality metrics across all samples were aggregated with MultiQC. Read trimming, adapter removal, and overall quality filtering were performed using FastP, after which high‐quality reads were assembled with SPAdes v4.2.0. Resulting scaffolds were annotated using Prokka v1.14.5 to predict coding sequences and functional elements.

**Figure 1:**
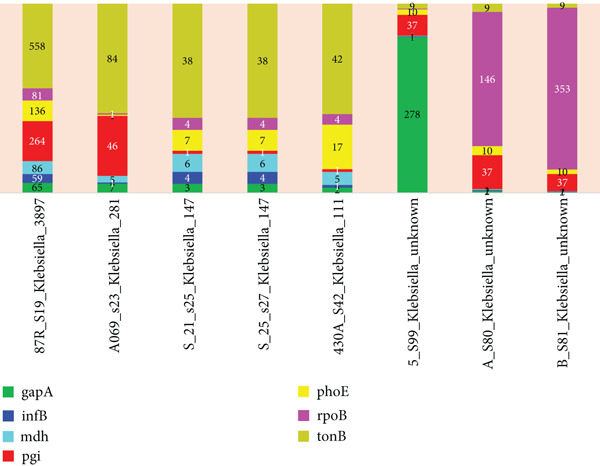
Genetic relatedness of the *K. pneumoniae* isolates from different sources.

**Figure 2:**
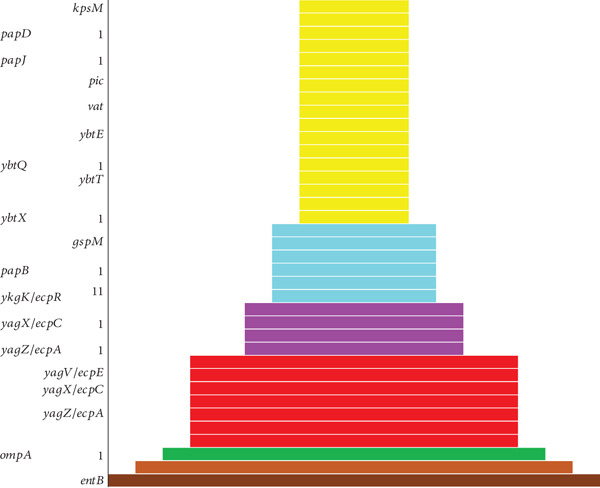
Comparing the virulent gene distribution of all samples from different sources.

To characterize virulence and antibiotic resistance determinants, annotated assemblies were screened with Abricate v1.0.1 against the VFDB, CARD, NCBI, and PlasmidFinder databases using default thresholds (≥ 90% identity and ≥ 80% minimum coverage). Multilocus sequence typing (MLST) was performed to assign sequence types and infer strain‐level relatedness among isolates (Figures 1 and 2) (Table 1).

**Table 1:** Strain identification using multilocus sequence typing (MLST).

**Strain**	**gapA**	**infB**	**mdh**	**pgi**	**phoE**	**rpoB**	**tonB**
*87R_S19_K. pneumoniae _3897*	gapA(65)	infB(59)	mdh(86)	pgi(264)	phoE(136)	rpoB(81)	tonB(558)
*A069_s23_K. pneumoniae _281*	gapA(7)	infB(1)	mdh(5)	pgi(46)	phoE(1)	rpoB(1)	tonB(84)
*S_21_s25_K. pneumoniae _147*	gapA(3)	infB(4)	mdh(6)	pgi(1)	phoE(7)	rpoB(4)	tonB(38)
*S_25_s27_K. pneumoniae _147*	gapA(3)	infB(4)	mdh(6)	pgi(1)	phoE(7)	rpoB(4)	tonB(38)
*430A_S42_K. pneumoniae _111*	gapA(2)	infB(1)	mdh(5)	pgi(1)	phoE(17)	rpoB(4)	tonB(42)
*5_S99_K. pneumoniae _113*	gapA(~278)	infB(1)	mdh(1)	pgi(37?)	phoE(10)	rpoB(1)	tonB(9)
*A_S80_K. pneumoniae _121*	gapA(2)	infB(1,118,123)	mdh(1)	pgi(37)	phoE(10)	rpoB(146)	tonB(9)
*B_S81_K. pneumoniae _215*	gapA(2)	infB(1,123)	mdh(1)	pgi(37)	phoE(10)	rpoB(353?)	tonB(9)

**Figure 3:**
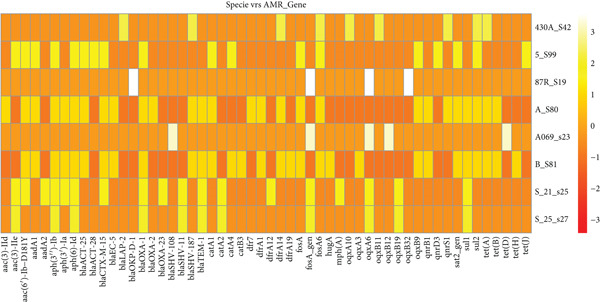
A heat map describing the strains of *K. pneumoniae* and their AMR genes. The figure is dominated by orange and yellow shades, indicating that most isolates harbor multiple AMR genes, but with varying intensities. White or very bright yellow spots represent highly abundant or strongly enriched AMR genes, whereas darker orange regions suggest lower counts or absence. The heat map demonstrates distinct, source‐specific resistome signatures among *K. pneumoniae*.

**Figure 4:**
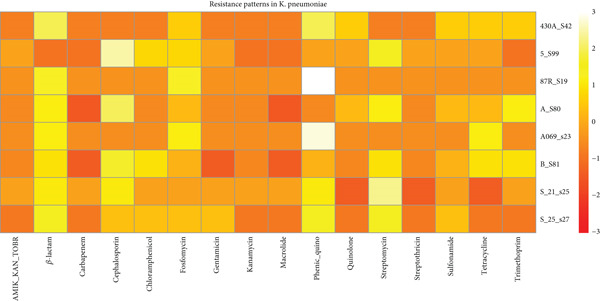
Heat map revealing that the resistance determinants in *K. pneumoniae* isolates are widely distributed across nearly all major antibiotic classes, with especially dense representation for *β*‐lactams, aminoglycosides, tetracyclines, and folate pathway inhibitors. Sparse representation in carbapenems and macrolides. The patterns indicate strong coselection and cocarriage of AMR genes.

**Figure 5:**
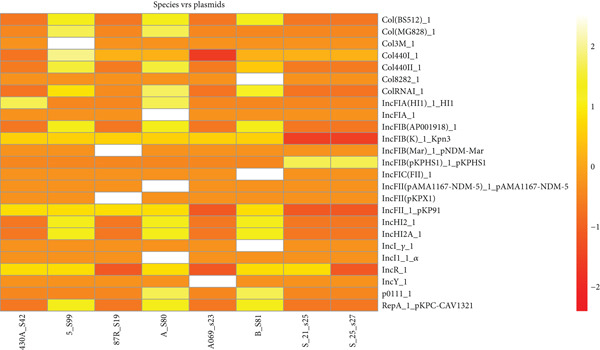
Heat map displaying the distribution of plasmid replicon types across *K. pneumoniae* isolates obtained from diverse ecological and clinical sources, including hospital wastewater, urinary tract infections, filarial wound infections, and cervical cancer samples. The *Y*‐axis represents the detected plasmids while the *X*‐axis corresponds to the individual bacterial isolates. Colour intensities reflect scaled abundance or detection frequency, where bright yellow indicates high plasmid signal, orange represents moderate presence, and white denotes absence.

Downstream analyses incorporated outputs from the above tools to interrogate AMR gene content, gene prevalence, genomic context, and comparative metrics. Data manipulation and visualization were conducted using R (v4.4.0), Unix command‐line utilities, and Microsoft Excel for tabular preprocessing, including extraction of sequence length, coverage metrics, genomic coordinates, and AMR gene frequency and plasmids distributions (Figures 3, 4, and 5). Sample relatedness was additionally visualized using Google Sheets (Figure 1).

**Figure 6:**
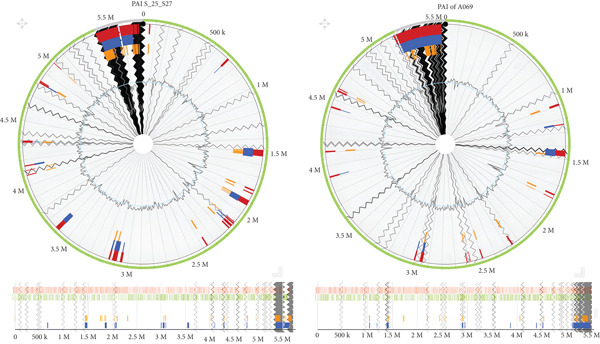
The pathogenicity islands of *K. pneumoniae* isolated from UTI and wound infection in Ghana, respectively, aligned against the reference genome *K. pneumoniae* 342, complete genome. The red or orange regions detected by SIGI‐HMM, high‐confidence pathogenicity islands, detect GIs based on codon usage differences. The blue or green regions detected by IslandPath‐DIMOB. Other genomic islands, including antibiotic resistance islands, identify regions with mobility genes (e.g., integrases and transposases) and tRNA gene associations. Gray or background regions: core genome sequences, not predicted as islands. IslandPick compares closely related genomes to detect unique foreign DNA. The zigzag lines are the contig boundaries. The outer boundary is alignment.

**Figure 7:**
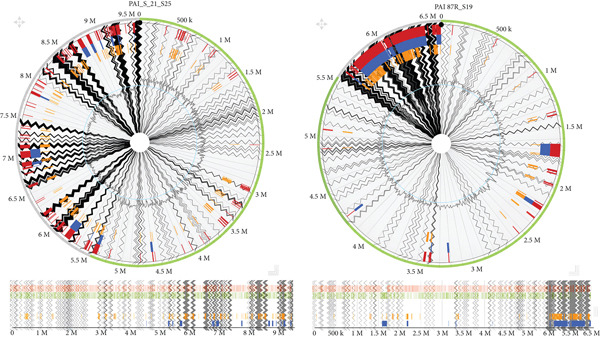
The pathogenicity island of *K. pneumoniae* isolated from UTI and hospital wastewater in Ghana, respectively, aligned against the reference genome *K. pneumoniae* 342, complete genome. The red or orange regions detected by SIGI‐HMM, high‐confidence pathogenicity islands, detect GIs based on codon usage differences. The blue or green regions detected by IslandPath‐DIMOB. Other genomic islands, including antibiotic resistance islands, identify regions with mobility genes (e.g., integrases and transposases) and tRNA gene associations. Gray or background regions: core genome sequences, not predicted as islands. IslandPick compares closely related genomes to detect unique foreign DNA. The zigzag lines are the contig boundaries. The outer boundary is alignment.

**Figure 8:**
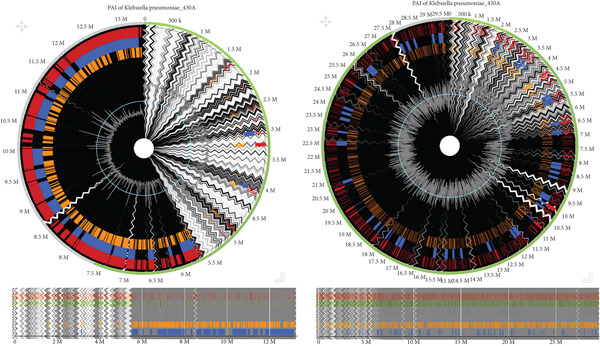
The pathogenicity island of *K. pneumoniae* isolated from a cervical cancer patient in Togo and UTI in Benin, respectively, aligned against the reference genome *K. pneumoniae* 342, complete genome. The red or orange regions detected by SIGI‐HMM, high‐confidence pathogenicity islands, detect GIs based on codon usage differences. The blue or green regions detected by IslandPath‐DIMOB. Other genomic islands, including antibiotic resistance islands, identify regions with mobility genes (e.g., integrases and transposases) and tRNA gene associations. Gray or background regions: core genome sequences, not predicted as islands. IslandPick compares closely related genomes to detect unique foreign DNA. The zigzag lines are the contig boundaries. The outer boundary is alignment.

**Figure 9:**
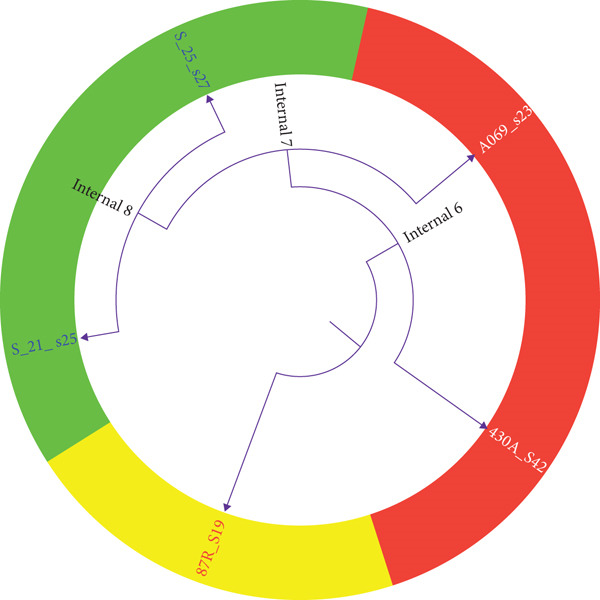
A recombination‐corrected phylogenetic tree showing a more accurate clonal relationship in *K. pneumoniae* strains isolated from UTI. Trees were generated using Itol. A recombination‐corrected phylogenetic tree is a type of evolutionary tree that shows the relationships between bacterial strains after removing the effects of recombination. The internal nodes serve as the lineages or common ancestor to the samples on the leaves.

**Figure 10:**
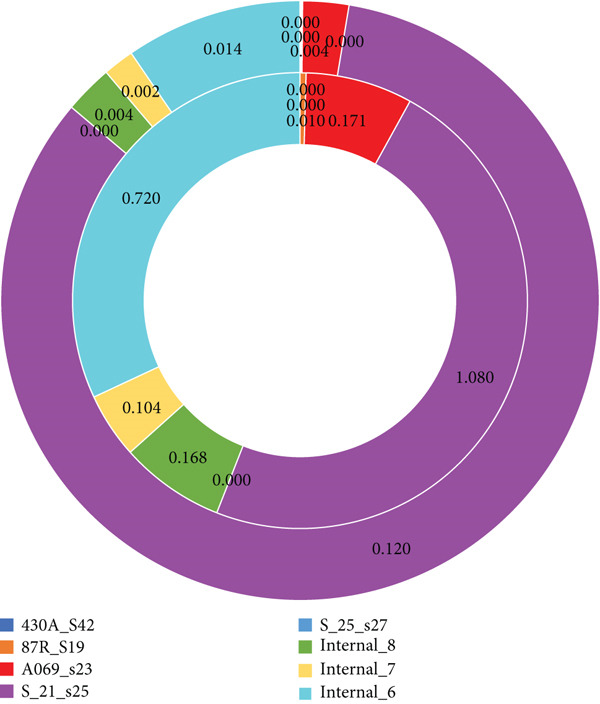
Doughnut plot showing *r*/*m* versus rho/theta. Inner ring: *r*/*m* is the recombination/mutation ratio which is the ratio of substitutions introduced by recombination versus mutation. Greater than one means that the recombination is a stronger driver of diversity than mutation. Less than one means that the mutation is dominant. The outer ring rho/theta stands for the rate of recombination relative to mutation events. Often very low, but it gives a sense of how frequently recombination occurs compared to point mutation.

**Figure 11:**
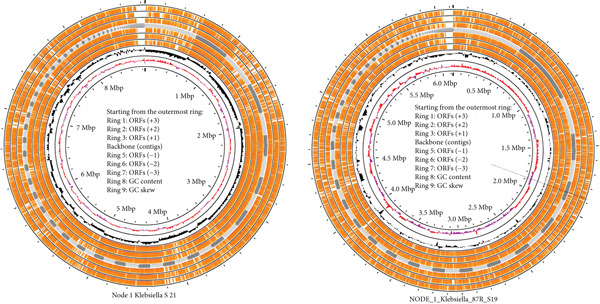
Open read frames of NODE_1 *K. pneumoniae* from UTI and hospital wastewater. The outermost rings (orange) represent the predicted ORFs in all six reading frames (+1, +2, +3, −1, −2, and −3). The black ring shows the assembled contigs forming the genomic backbone. Ring 8 is the GC content. GC skew is Ring 9 in magenta coloration which reveals replication origin (*oriC*) and terminus (*terC*).

Genomic island prediction was performed using IslandViewer 4, integrating multiple prediction algorithms to identify pathogenicity and resistance‐associated islands (Figures 6, 7, and 8). Recombination analysis was conducted with Gubbins in combination with ClonalFrameML to estimate SNP‐based phylogenies, recombination events, and *r*/*m* ratios (Figures 9 and 10 and Table 2). Proksee v2.0 was used to generate genome maps and evaluate open reading frame (ORF) organization and structural genome features (Figure 11).

**Table 2:** Core‐gene alignment per branch statistics.

***K. pneumonia* samples**	**Total SNPs**	**No. of SNPs inside recomb**	**No. of SNPs outside recomb**	**No. of recomb blocks**	**r**/**m**	**rho/theta**
*430A_S42*	0	0	0	0	0.000	0.000
*87R_S19*	172,079	1674	170,405	36	0.010	0.000
*A069_s23*	10,526	1539	8987	32	0.171	0.004
*S_21_s25*	52	27	25	3	1.080	0.120
*S_25_s27*	0	0	0	0	0.000	0.000
*Internal_8*	10,869	1565	9304	35	0.168	0.004
*Internal_7*	11,919	1125	10,794	26	0.104	0.002
*Internal_6*	13,927	5831	8096	111	0.720	0.014
*Internal_ROOT*	0	0	0	0	0.000	0.000

## 3. Results

This study titled *“*Antimicrobial Resistant Factors in *K. pneumoniae* Strains Isolated From Urinary Tract Infections, Wound Infections, and Cervical Cancers from Ghana, Togo, and Benin” is aimed at exploring genomic AMR determinants and other genomic features of *K. pneumoniae* isolated from different sources to ascertain the enabling genetic elements of the bacteria against antibiotics and possible variation in response to effects of antibiotics with respect to the source of isolation. As a result, eight samples were isolated from UTI, wound infection, cervical cancers, and hospital environment for the study.

### 3.1. Identification and Relatedness of the *K. pneumoniae* Species

After genome assembly, species identification was necessary to know the exact bacterial species being sampled as well as identifying their housekeeping genes using MLST. MLST is a technique used in molecular biology to classify bacterial strains or other microorganisms by analyzing the sequences of multiple housekeeping genes that are conserved and essential for the organism’s basic functions. Table 1 summarizes the MLST results from our study.

From Table 1, the first column is the strains, while the rest of the columns are the housekeeping genes. Strains *87R_S19_K. pneumoniae _3897*, *A069_s23_K. pneumoniae _281*, *S_21_s25_K. pneumoniae _147*, *S_25_s27_K. pneumoniae _147*, *430A_S42_K. pneumoniae _111*, *5_S99_K. pneumoniae _113*, *A_S80_K. pneumoniae _121*, and *B_S81_K. pneumoniae _215* were isolated from hospital wastewater (Gh), wound infection (Gh), UTI (Gh), cervical cancer patient (Togo), and UTI (Benin). From MLST official GitHub page, the following parameters are explained: n means the exact intact allele; ~n means the novel full‐length allele similar to n 100% ≥ ‐‐minid; n? means the partial match to known allele; ‐ means allele missing; and n,m means multiple alleles.

The figure shows a stack bar graph of the *K. pneumoniae* genetic relatedness. Each stack shows the allele identity number while each bar represents the species from specific isolation source. Species with the same allele identity numbers on each stack are deemed related.

Both the analysis of the genomic sequences in Table 1 and relatedness in Figure 1 did not only identify the sample species but also examine their genetic relatedness. This approach was crucial, as genes located in close proximity on the genome are often inherited together and may contribute collectively to virulence. The analysis found that while all the samples shared the same housekeeping genes, their allele identity numbers varied. Specifically, Samples S_21_S25 and S_25_S27 from UTI in Ghana exhibited identical allele identity numbers across all housekeeping genes, suggesting a close genetic relationship [13]. In contrast, the other samples from diverse sources as mentioned already displayed different allele numbers, indicating genetic divergence. These findings provide insights into the evolutionary relationships among the strains and their potential implications for antimicrobial resistance and pathogenicity [14].

### 3.2. Virulence Gene Analysis of *K. pneumoniae*

*K. pneumoniae* possesses several virulence genes that contribute to its ability to cause infections, predominantly in immunocompromised persons and those working in hospital settings [15] which enhance its ability to cause infections, particularly in immunocompromised individuals and people in the healthcare settings. In this study, the number of virulence genes was determined for each sample, and notably, some of the virulent genes such as *entB*, *entA*, and *ompAI* dominated the others in terms of quantity as reported already in other studies [16]. Figure 2 summarizes the virulent factors this study has identified in the bacterial samples.

The right side of Figure 2 shows the selected virulence genes, and the right side of the funnel chart shows each bar corresponding to a sample, while the length of the bars shows the number of virulence genes in that sample. The bars in yellow colours are the virulence genes of sample S_21_s25; bars in cyan colours: virulence genes of sample 5_S99; bars in magenta colours: virulence genes of samples A_S80 and A_S81; bars in green colours: virulence genes of sample 430A_S42; and bars in brown colours: virulence genes of sample S_25_s27.

### 3.3. Antibiotic Resistance Patterns in *K. pneumoniae*

*K. pneumoniae* employs varied mechanisms to exert antibiotic resistance including beta‐lactamase production that produces extended‐spectrum beta‐lactamases and AmpC beta‐lactamases, leading to resistance against penicillin, cephalosporins, and monobactams; carbapenem resistance through carbapenemase such as *KPC*, *NDM*, and *VIM* rendering carbapenems ineffective; efflux pumps that remove antibiotics from bacterial cells, reducing drug efficacy; porin loss or modification that alters membrane permeability, limiting antibiotic entry; and plasmid‐mediated resistance that happens by acquiring resistance genes through horizontal gene transfer [17]. Figures 3 and 4 summarize the resistant patterns noted in this study.

Besides the virulence genes, *K. pneumoniae* is a known opportunistic pathogen branded for its significant antibiotic resistance, which poses challenges in treatment [18] of its infections. This study, as seen in Figure 4, identified multiple resistance patterns alongside other genomic features of the bacteria. One notable pattern was triple therapy resistance against amikacin, kanamycin, and tobramycin [19], observed in some of the samples such as 5_S99, A_S80, and B_S81, which were isolated from a UTI in Benin. This resistance was noted to be mediated by the *aac(6* ^′^ *)-Ib-D181Y* gene and its subtypes, which were initially identified by [20] as shown in Figure 3, enabling the bacteria to counteract the effects of these antibiotics.

Another detected resistance mechanism was beta‐lactam resistance, which involves genes encoding beta‐lactamase enzymes that degrade and inactivate beta‐lactam antibiotics. The identified resistance genes included *blaSHV-187*, *blaLAP-2*, *blaTEM-1*, *blaSHV-187*, *blaOXA-2*, *blaTEM-1*, *blaSHV-187*, and *hugA*, distributed across the samples from Benin (A_S80 and B_S81) and Too (430A_S42) at various loci. This diverse distribution of these resistance genes suggests that *K. pneumoniae* employs a complex and multifaceted mechanism to evade antibiotic treatment, as was also reported by [21].

Additional resistance patterns are also observed in Figure 4: Cephalosporin resistance [22] can be seen in Samples 5_S99, A_S80, and B_S81, while fosfomycin resistance is noted in Samples 5_S99, A_S80, B_S81, and 430A_S42 facilitated by the *fosA* gene. Gentamicin resistance is observed in 5_S99 from Benin, mediated by the *aac(3)-IIe* gene located on the negative strand of the genome [23].

Furthermore, streptomycin resistance can also be observed in Samples 5_S99, A_S80, and B_S81, primarily conferred by different derivatives of *aph* and *aadA1* genes [24], which were predominantly located on the positive strand, which was isolated from a UTI in Benin. Notably, in Sample A_S80, an additional resistance mechanism is observed, where the bacteria employed a combination of *aph(3* ^″^ *)-Id*, *aph(3* ^″^ *)-Ib*, and *AadA1* genes to confer resistance. These findings highlight the diverse genetic strategies utilized by *K. pneumoniae* to resist multiple classes of antibiotics.

A relatively novel resistance pattern observed in the study was phenicol–quinolone resistance in Samples A_S80 and 5_S99 from UTI Benin, driven by the AMR genes (*qnrB1* and *qnrS1*) and *qnrD3*, respectively. These genes are detected at different locus positions but maintained the same sequence length, suggesting their fixation across all strains of the species. Additionally, low resistance patterns included sulfonamide, trimethoprim, tetracycline, chloramphenicol, quinolone, and methicillin resistance across most of the samples, still portraying the diverse mechanisms the bacteria adopt in evading antibiotic effects.

### 3.4. Plasmids

Following the AMR genes detected in the study, there is a need to explore plasmids across the sample species and their association with the AMR genes. Basically, plasmids are circular small pieces of DNA structures noted for moving antibiotic resistance genes between bacterial species through a process called horizontal gene transfer [25]. Plasmids play a dangerous role in antimicrobial resistance by providing conduits for gene transfer among bacteria. In this study, a number of plasmids have been identified in some of the *K. pneumoniae* strains and are summarized in Figure 5.

An important component of this study was the exploration of plasmids within the bacterial genomes. Plasmids play a crucial role in the emergence and dissemination of AMR among bacteria, functioning as mobile genetic elements that carry and transfer resistance genes across cells. In this study, all isolates were found to contain plasmids as shown in Figure 5, though the number and types varied across samples [26].

Notably, Samples 5_S99, A_S80, and B_S81, which were isolated from UTIs in Benin, harbored a greater abundance of *Col* and *Inc* plasmid groups, particularly *Col440I_1* and *IncF* [27] as observed in Figure 5. In contrast, the isolate 430A_S42, obtained from a cervical cancer case in Togo, did not possess any *Col* plasmids. Interestingly, isolates from UTI cases in Ghana exhibited a different plasmid distribution pattern compared to those from Benin, suggesting possible geographic or clinical variations in plasmid prevalence and mobility.

Among the plasmid types, the *Inc* group is notably the second most predominant after the *Col* group. Inc plasmids are characterized by their ability to coexist with other plasmids within a single bacterial cell and are often associated with multidrug resistance (MDR). Within this group, the study identified *IncF* plasmid, a large conjugative plasmid commonly found in *E. coli* and *IncH* plasmids, which are linked to resistance against heavy metals and multiple antibiotics, particularly in *Salmonella* species.

The most dominant plasmid category observed is the *Col* plasmids. These plasmids are central to bacterial competition and survival, as they encode genes for colicins, protein toxins that kill or inhibit closely related bacterial strains, thereby conferring a competitive advantage to the host bacterium. The *Col* plasmids detected, particularly *ColRNAI*, have been extensively characterized for their roles in bacterial population dynamics and horizontal gene transfer [28]. *Col* plasmids are generally small and can either exist independently or integrate additional genetic elements, including AMR genes.

Interestingly, *K. pneumoniae* isolates that appeared phylogenetically related, such as S_21_S25 and S_25_S27, did not share identical AMR determinants in terms of plasmid content and resistance profiles. This observation as shown in the figure suggests that even closely related strains may acquire or lose plasmids independently, reflecting the dynamic nature of plasmid‐mediated resistance evolution.

### 3.5. Exploring PAI of *K. pneumoniae*

PAIs are distinct genetic elements in bacterial genomes that contribute to virulence. They are large chromosomal or plasmid‐associated DNA regions that contain clusters of genes responsible for bacterial pathogenicity [29]. These genes encode factors such as toxins, adhesion molecules, secretion systems, and antibiotic resistance. The key features of PAIs include G + C content variation which suggests horizontal gene transfer; presence of mobility genes such as insertion sequences, transposases, or integrases, indicating their ability to transfer between bacteria; and association with tRNA genes closer to tRNA genes, serving as integration sites [30]. Other unique features of PAI are its unstable nature due to its mobile elements; PAIs can be lost or acquired, influencing bacterial virulence and adaptation. In general, PAI contributes a lot to pathogenesis through virulence factors like Type III and Type IV secretion systems, toxins like Shiga toxin, and adhesion proteins. Figures 6, 7, 8, 9, 10, and, 11, summarize the PAIs and Gis of the *K. pneumoniae* strains explored in this study.

The second major focus of this study was the characterization of PAIs within the bacterial genomes. In this study, Sample S_21_S25 exhibited a notably high number of genetic islands (GIs), occurring approximately every 500 kb along the genome. This dense distribution suggests that the isolate has a high capacity to acquire and maintain virulence‐related genetic determinants. Within the same species, nine distinct regions were also identified as “other GIs.” Interestingly, the first four GIs were located within the initial 1.7 Mb region of the genome, while the remaining ones were positioned near the terminal regions. This positional bias indicates that plasmids, AMR genes, integrases, transposases, and tRNA genes may be preferentially concentrated toward the genome’s end.

Conversely, the IslandPick model, which identifies unique foreign DNA by comparing closely related genomes, did not detect any foreign genetic material within this isolate. This finding suggests limited horizontal gene transfer or recent acquisition of foreign DNA in this particular genome. Such genomic stability may imply that the virulence and resistance traits are evolutionarily conserved within the species rather than recently introduced.

When comparing the distribution of PAIs across different isolates including A069_S23, S_25_S27, S_21_S25, and 87R_S19, a marked diversity in GI content and structure is observed. These variations reflect differing virulence potentials and genomic plasticity among isolates of *K. pneumoniae* from various clinical sources. Also, even genetically related isolates did not exhibit identical pathogenicity profiles as was also reported by, emphasizing the complexity and dynamic nature of the *K. pneumoniae* genome, driven by recombination, horizontal gene transfer, and selective adaptation to distinct host environments.

### 3.6. Genome Recombination Analysis

A recombination‐corrected phylogenetic tree is a refined version of a traditional phylogenetic tree that accounts for the confounding effects of recombination events in bacterial genomes [31]. In bacteria, genetic recombination particularly horizontal gene transfer can introduce DNA segments from unrelated strains or species [32]. These segments may cause unrelated strains to appear closely related in a phylogenetic analysis if not properly accounted for. As a result, a tree constructed from uncorrected genomic data may misrepresent the true evolutionary relationships between strains. To overcome this, the bioinformatics tool Gubbins was used to identify and mask recombinant regions in the *K. pneumoniae* genomes alignment before tree construction. The resulting recombination‐corrected tree as shown in Figure 4 is based only on genetic variations that are most likely due to vertical inheritance (mutation), providing a more accurate view of clonal relationships. This correction is especially important in bacterial population studies where horizontal gene transfer is common, as it ensures that the phylogeny reflects true ancestry rather than normal genetic exchange events (Figures 9 and 10 and Table 2).

From Table 2, Column 1 shows *K. pneumoniae* samples **(**e.g., *430A_S42* and *87R_S19*) and internal nodes in the phylogeny (*internal_6*, *internal_7*, and *ROOT*). Column 2 shows the total number of single nucleotide polymorphisms detected relative to the reference. Column 3 shows SNPs that fall within predicted recombination regions. Column 4 shows SNPs attributed to clonal inheritance (vertical evolution). Column 5 shows the number of distinct recombination events detected. Column 6 shows the (recombination/mutation ratio) ratio of substitutions introduced by recombination versus mutation. Column 7 shows rho/theta, the rate of recombination relative to mutation events. Often very low, but it gives a sense of how frequently recombination occurs compared to point mutation.

### 3.7. Interpretation of the Entire Recombination Results

From the recombination analysis presented in Table 2, clear differences in the evolutionary dynamics of the *K. pneumoniae* isolates were observed. A subset of isolates, such as 430A_S42 and S_25_s27, recorded zero SNPs and no evidence of recombination, suggesting that they are highly clonal or identical to the reference genome. In contrast, isolate 87R_S19 exhibited an extremely high SNP count (172,079), with only a small fraction associated with recombination, resulting in a very low *r*/*m* ratio (0.01). This indicates that mutations are the dominant force shaping its genome [26]. Similarly, isolates A069_S23 and internal_8 displayed moderate SNP counts, with approximately 10%–15% of SNPs found within recombination regions (*r*/*m* ≈ 0.17), pointing to a mixed contribution of mutation and recombination, though mutation remained the primary driver. Interestingly, isolate S_21_S25 presented a low SNP count in all, but with more SNPs inside recombination blocks than outside (27 vs. 25), giving an *r*/*m* value above 1.0. This suggests that recombination, rather than mutation, is the dominant evolutionary mechanism in this isolate [27]. The lineage represented by internal_6 stood out as highly recombinogenic, with 13,927 SNPs and a substantial proportion located within recombination regions (*r*/*m* = 0.72), consistent with frequent horizontal gene transfer events.

The analysis of rho/theta ratios further supports these findings, with generally low values (0.002–0.014) across isolates, indicating that mutation events occur more frequently than recombination. However, when recombination does occur, it tends to introduce a larger number of SNPs simultaneously, amplifying its evolutionary impact. Notably, S_21_S25 deviated from this trend, with a relatively higher rho/theta value (0.12), reflecting a more recombination‐driven evolutionary process. Examination of internal nodes revealed that internal_6, in particular, emerged as a highly recombinogenic ancestral lineage, likely playing a key role in shaping downstream diversity. Internal_7 and internal_8 also exhibited moderate recombination activity, highlighting their contribution to the broader evolutionary dynamics.

Overall, the findings suggest that mutation is the predominant driver of genetic diversification among most *K. pneumoniae* isolates, particularly in highly mutational lineages such as 87R_S19 and A069_S23. However, certain isolates and lineages, notably S_21_S25 and internal_6, appear to have been shaped more profoundly by recombination, underscoring the role of horizontal gene transfer in accelerating genetic change. Conversely, the near‐clonal isolates (430A_S42 and S_25_S27) illustrate the persistence of highly conserved lineages within the population. Together, these results reveal a mixed evolutionary landscape in *K. pneumoniae*, where mutation generally dominates, but recombination serves as a powerful force in specific lineages, creating genetic “hotspots” that may facilitate adaptation and diversification.

### 3.8. ORF Analysis of *K. pneumoniae*

Insights into genome organization, coding potential, and features are relevant to AMR, and one way to assess these features of *K. pneumoniae* is by ORF. An ORF is a continuous sequence of DNA or RNA that has the potential to be translated into a protein [13]. In bacterial genomes, ORFs provide essential genetic information for protein‐coding genes, virulence factors that encode toxins, adhesion proteins, or secretion systems that enhance bacterial pathogenicity; antibiotic resistance genes encoding for resistance mechanisms like beta‐lactamases, efflux pumps, or modified drug targets; and horizontal gene transfer elements such as mobile genetic elements like plasmids and transposons which facilitate gene transfer between bacteria. In this study, we have explored the ORFs of the NODE_1 of all the *K. pneumoniae* samples from different sources, and the results are visualized in Figure 11.

The ORF maps of *K. pneumoniae* strains (Figure 11) highlight their gene‐dense genomes, with ORFs evenly distributed across all six reading frames, indicating few noncoding gaps. The contig backbone is largely continuous, reflecting a high‐quality assembly, while observed discontinuities point to possible plasmid or mobile genetic element insertions [14]. Genomic variation is evident in the GC content profile, where sharp deviations from the average GC% suggest the presence of genomic islands. These regions are known hotspots for horizontally acquired genes, often encoding AMR determinants, virulence factors, or metabolic traits. The GC skew further revealed replication origins and termini, with irregularities suggesting plasmid integration or other foreign DNA acquisition events. Collectively, these genomic features emphasize the high coding potential and remarkable adaptability of *K. pneumoniae*. The presence of atypical GC content regions and mobile element signatures underscores the role of horizontal gene transfer in shaping its resistome [33]. This supports the organism’s well‐established reputation as a multidrug‐resistant pathogen, with resistance islands and plasmid‐borne cassettes likely contributing to the dissemination of key resistance genes such *as blaKPC*, *blaNDM*, and *ESBLs*.

## 4. Discussion

This study provides a high‐resolution genomic reconstruction of *K. pneumoniae* circulating across diverse ecological niches in West Africa and highlights the organism’s remarkable evolutionary flexibility. By combining MLST profiling, virulence and AMR gene characterization, genomic island mapping, ORF architecture, variant analysis, and recombination modelling, we delineate the multiple layers of genomic processes that collectively shape the pathogenicity and antimicrobial resistance of *K. pneumoniae*. The coexistence of clonal strains and highly divergent lineages within the same geographical region underscores a dynamic population structure in which both vertical inheritance and horizontal acquisition drive genomic diversification. The observation that UTI‐associated strains in Ghana share identical allele identity numbers indicates recent expansion of a successful clone, possibly reflecting a stable host‐associated lineage or local outbreak. In contrast, the divergence observed in several isolates from wastewater and wounds suggests long‐standing adaptive evolution influenced by environmental stress, antibiotic gradients, and interspecies interactions.

The virulence architecture of the isolates further reflects differential evolutionary trajectories. The dominance of genes such as *entA*, *entB*, *eсpA*, *eсpC*, *eсpR*, and *ompAI* illustrates the conserved importance of iron sequestration, adherence, and outer membrane integrity in successful host colonization [18]. The widespread presence of PAIs and GIs, especially in terminal genome regions, provides evidence of evolutionary hotspots where integrases, transposases, and tRNA‐flanking sequences facilitate the integration and excision of foreign DNA. These regions serve as genomic “ports of entry,” enabling the rapid assimilation of virulence‐associated loci and supporting the emergence of hypervirulent or high‐risk clones as was also reported by [19].

The AMR landscape revealed by this study highlights the extensive genomic investment *K. pneumoniae* makes in resisting antibiotic pressure. All isolates carried MDR genes as was also reported by [20], with UTI strains from Benin displaying a consistent triple resistance pattern against amikacin, kanamycin, and tobramycin, mediated by aminoglycoside‐modifying enzymes. The copiousness of multiple *bla* gene variants including *blaSHV*, *blaTEM*, *blaOXA*, and *blaLAP* demonstrates the bacteria’s ability to accumulate and maintain multiple *β-lactamase* systems, increasing redundancy and resilience under antibiotic exposure [21]. The universal presence of *fosA* highlights a worrying trend, as fosfomycin is often recommended for UTI treatment in Africa. Furthermore, the linkage of quinolone resistance to the *oqxA10* and *oqxB9* efflux operon indicates plasmid‐mediated dissemination, reinforcing the role of conjugative plasmids as efficient vehicles for interstrain and interspecies spread. The ORF architecture, marked by GC shifts and atypical gene density, strongly indicates the integration of plasmids, translocatable resistance cassettes, and potentially carbapenemase‐associated elements such as *blaKPC* and *blaNDM*. These structural signatures underscore *K. pneumonia*’s capacity for rapid genome remodeling in response to antibiotic pressure [22].

From an evolutionary standpoint, the interplay between mutation and recombination varied across the strains and revealed the mechanisms underlying adaptive potential. Clonal isolates, primarily from cervical cancer from Togo and UTI cases in Ghana (Samples 430A_S42 and S_25_s27), exhibited no SNP variation and lacked recombination signals, reflecting highly stable lineages likely maintained through vertical inheritance [23]. Their genomic stability may indicate selective advantages within a specific niche, such as persistent colonization of the urinary tract. Conversely, isolates with high SNP counts but minimal recombination such as those from wastewater reflect mutation‐driven diversification, consistent with environmental bacteria exposed to fluctuating selective pressures and antibiotic residues [24]. Importantly, recombination‐dominated instances (e.g., S_21_S25 and internal_6 lineage) demonstrated high *r*/*m* ratios, indicating that a single recombination event introduced more SNPs than multiple independent mutations combined. These recombination‐driven hotspots represent evolutionary accelerators capable of reshaping large genomic segments, introducing novel AMR determinants, and generating emergent hybrid lineages with enhanced fitness as was reported by [26].

The overall low *ρ*/*θ* values observed (0.002–0.014) indicate that mutation remains the primary source of genetic diversification across most *K. pneumoniae* strains. However, the elevated *r*/*m* ratios in specific lineages highlight episodic bursts of recombination that can dramatically alter genomic composition in short evolutionary timeframes. This balance between mutation‐driven stability and recombination‐driven innovation supports a dual adaptive strategy: long‐term persistence via conserved genomic cores and rapid adaptability via mobile genetic elements and recombination events. Such a strategy enhances the ability of *K. pneumoniae* to thrive across clinical, environmental, and host‐associated niches [27].

In conclusion, these findings highlight the profound genomic plasticity of *K. pneumoniae*, driven by a tightly interwoven network of mutation, recombination, horizontal gene transfer, and plasmid integration. This plasticity underpins the bacteria’s capacity to evolve MDR and heightened virulence, posing a significant challenge to public health systems across West Africa and globally. The strong association between lineage structure, virulence composition, and AMR gene portfolios underscores the importance of genomic surveillance and integrated molecular diagnostics. Continuous monitoring of recombination hotspots, resistance islands, and emerging clonal lineages is essential for early detection of high‐risk strains and for informing targeted antibiotic stewardship strategies. The mechanistic insights provided in this study advance our understanding of *K. pneumoniae* evolution and outline critical genomic features that may serve as future targets for therapeutic intervention, vaccine development, and molecular tracking of the AMR menace, especially in Africa.

**Nomenclature**
AMR: antimicrobial resistance
GC: guanine cytosine
AT: adenine–thymine
MLST: multilocus sequence typing
gbk: gene bank
gff: general feature format
Inc: incompatibility
Col: colicinogenic
ORF: open reading frame
PAI: pathogenicity island
UTI: urinary tract infection
SEQ_LEN: sequence length
GENE_SEQ: gene sequence
GI: genetic island
RBS: ribosome binding site
WHO: World Health Organization
DNA: deoxyribonucleic acid
RNA: ribonucleic acid

## Ethics Statement

All ethical approvals were obtained by the respective research laboratories and centers who provided us with the samples duly acknowledged.

## Consent

Consent for publications was obtained from the participants before the samples were taken.

## Disclosure

The author has perused and agreed with the content of the manuscript. The author certifies that the submission is original work and is not under review at any other publication. This study declares the following information as valid and true.

## Conflicts of Interest

The author declares no conflicts of interest.

## Author Contributions

Biigba Yakubu, the corresponding author, conceptualized the study idea, did the entire wet lab processes from antibiotic sensitivity testing, DNA extraction, library preparation, sequencing, analysis, and writing of the manuscript.

## Funding

No funding was received for this manuscript.

## General Statement

*Limitations to the Study*. The limitation of the study was the limited number of samples which hindered extensive comparative analysis and broad‐based conclusions from the findings.

## Data Availability

All the data used in this study can be obtained from the author upon request via email.
